# The Metric System

**Published:** 1879-06

**Authors:** 


### THE METRIC SYSTEM.

The following simple table, says the *Boston Transcript*, gives all that there is in the metric or decimal system of weights and measures:

MONEY.

10 mills make a cent.

10 cents make a dime.

10 dimes make a dollar.

10 dollars make an eagle.

LENGTH.

10 milli-meters make a centimeter.

10 centi-meters make a decimeter.

10 deci-meters make a meter.

10 *meters make a decameter.

10 deca-meters make a hectometer.

10 hecto-meters make a kilometer.

10 kilo-meters make a myriameter.

WEIGHT.

10 milli-grammes make a centigramme.

10 centi-grammes make a decigramme.

10 deci-grammes make a gramme.

10 †grammes make a decagramme.

10 deca-grammes make a hectogramme.

10 hecto-grammes make a kilogramme.

10 kilo-grammes make a myriagramme.

CAPACITY.

10 milli-liters make a centiliter.

10 centi-liters make a deciliter.

10 deci-liters make a liter.

10 ‡liters make a decaliter.

10 deca-liters make a hectoliter.

The square and cubic measures are nothing more than the

*A meter is equal to 39.368 American inches.
†A gramme is equal to 15.433 grains troy or avoirdupois.
‡A liter is equal to 2.113 American pints.

squares and cubes of the measure of length. (Thus a square and a cubic millimeter are the square and the cube of which one side is a millimeter in length). The *are* and *stere* are other names for the square dekameter and the cubic meter. Or the following table may make it more plain to some.

METRIC MEASURES OF LENGTH.

| | | | | |
|---|---|---|---|---|
| 1 Millimetre | 0.001 | equals | .039 | inches |
| 1 Centimetre | 0.01 | " | .393 | " |
| 1 Decimetre | 0.1 | " | 3.937 | " |
| 1 METRE | 1. | " | 39.370 | " |
| 1 Kilometre | 1000. | " | .62 | miles |

APPROXIMATE EQUIVALENTS.

| | | | |
|---|---|---|---|
| 1 *m* or 1 gr. | equals | .06 | grams |
| 1 f ʒ or 1 ʒ | " | 4. | " |
| 1 f ℥ | " | 30. | " |
| 1 ℥ | " | 31. | " |
| 1 f ℥ Glycerine | " | 37. | " |
| 1 f ℥ Syrups | " | 40. | " |

METRIC WEIGHTS.

| | | | | |
|---|---|---|---|---|
| 1 Milligram | 0.001 | equals | $\frac{1}{64}$ | gr. |
| 1 Centigram | 0.01 | " | $\frac{1}{6}$ | " |
| 1 Decigram | 0.1 | " | $1\frac{1}{2}$ | " |
| 1 GRAM | 1. | " | 15.432 | |
| 1 Kilogram | 1000. | " | 2.7 | lb. |

TEMPERATURE.

| | | |
|---|---|---|
| 37° Cent. | 98°.6 | Fahr. |
| 38° " | 100°.4 | " |
| 39° " | 102°.4 | " |
| 40° " | 104°. | " |
| 41° " | 105°.8 | " |

1° C. equals 1°.8 F. Multiply C. by 1.8, add 32 equals F.

